# Implementation of a national policy for improving health and social care: a comparative case study using the Consolidated Framework for Implementation Research

**DOI:** 10.1186/s12913-019-4591-2

**Published:** 2019-10-22

**Authors:** Helena Strehlenert, Johan Hansson, Monica Elisabeth Nyström, Henna Hasson

**Affiliations:** 1 Department of Learning, Informatics, Management and Ethics, Medical Management Centre, Karolinska Institutet, SE 171 77 Stockholm, Sweden; 2 Department of Public Health Analysis and Data Management, Public Health Agency of Sweden, SE 171 82 Solna, Sweden; 3 Department of Public Health and Clinical Medicine, Epidemiology and Global Health, Umeå University, SE 901 87 Umeå, Sweden; 4 Center for Epidemiology and Community Medicine, Stockholm County Council, SE 171 29 Stockholm, Sweden

**Keywords:** Policy, Implementation, Wicked problems, Elderly care, Consolidated framework for implementation research, CFIR, Large-scale change, National quality registry

## Abstract

**Background:**

Comprehensive policies are becoming common for addressing wicked problems in health and social care. Success of these policies often varies between target organizations. This variation can often be attributed to contextual factors. However, there is a lack of knowledge about the conditions for successful policy implementation and how context influences this process. The aim of this study was to investigate county-level actors’ perspectives on the implementation of a comprehensive national policy in three Swedish counties. The policy focused on developing quality of care for elderly based on the use of national quality registries (NQRs) and to improve coordination of care.

**Methods:**

A comparative case study approach was used. Data was collected longitudinally through documents and interviews. The Consolidated Framework for Implementation Research (CFIR) guided the analysis.

**Results:**

All three counties shared the view that the policy addressed important issues. Still, there was variation regarding how it was perceived and managed. Adaptable features—i.e., NQRs and improvement coaches—were perceived as relevant and useful. However, the counties differed in their perceptions of another policy component—i.e., senior management program—as an opportunity or a disturbance. This program, while tackling complex issues of collaboration, fell short in recognizing the counties’ pre-existing conditions and needs and also offered few opportunities for adaptations. Performance bonuses and peer pressure were strong incentives for all counties to implement the policy, despite the poor fit of policy content and local context.

**Conclusions:**

Comprehensive health policies aiming to address wicked problems have better chances of succeeding if the implementation includes assessments of the target organizations’ implementation capacity as well as the implicit quid pro quos involved in policy development. Special attention is warranted regarding the use of financial incentives when dealing with wicked problems since the complexity makes it difficult to align incentives with the goals and to assess potential consequences. Other important aspects in the implementation of such policies are the use of collaborative approaches to engage stakeholders with differing perspectives, and the tailoring of policy communication to facilitate shared understanding and commitment.

## Background

Comprehensive health policies are becoming common for addressing complex, overarching improvement needs, or wicked problems [1], in health and social care [2]. Such policies are often difficult to implement due to the complexity of the problems they aim to solve and to the complex organizational systems they address [3–5]. In wicked problems, the stakeholders involved have differing perspectives of the problem and the best ways to tackle it. Thus, wicked problems are difficult to define and are inherently resistant to unanimous solutions [1]. In addition, comprehensive policies are multifaceted and difficult to define, as they tend to change over time [6].

It is well-known that some of the variation in implementation outcomes can be attributed to contextual factors [7]. Current research suggests that the context is an active component in change and that it can be defined as a broad set of circumstances or unique factors that surround an implementation effort [8]. However, more research is needed to understand how different aspects of context interact with health policy content and the implementation process [9, 10]. Various taxonomies and frameworks are available for analyzing the interplay between an intervention, its processes and context. One such framework, the Consolidated Framework for Implementation Research (CFIR) [8], was used in this study. The CFIR was developed to facilitate the understanding of the wide range of constructs believed to influence implementation. The CFIR consists of five domains: 1) characteristics of the intervention, 2) outer setting, 3) inner setting, 4) characteristics of individuals and 5) the process of implementation. So far, the CFIR has been used across a variety of interventions, settings and units of analysis [11], but further investigation is needed to explore the use of CFIR to study less-specific interventions, such as comprehensive policy agreements [12, 13].

In 2010, the Swedish government launched a comprehensive national policy aiming to improve the quality and coordination of care for elderly with complex needs (“Agreement on coordinated health and social care for the most ill older people”) [14]. The policy was a “soft law”; i.e., it was a non-binding agreement between the central government and the politically governed interest organization for public organizations responsible for provision of health and social care (the Swedish Association of Local Authorities and Regions, SALAR).

The aim of the current study was to investigate key county level actors’ perspectives on the implementation of a comprehensive national policy in three counties in Sweden. The study contributes to the literature with knowledge about the formulation and implementation of comprehensive health policies addressing wicked problems. Furthermore, it contributes with increased understanding about how the CFIR can be used to study implementation of a comprehensive policy.

## Methods

### Design

A case study approach was used to compare the implementation of the policy in three counties in Sweden [15].

### The policy

The Swedish health care system is highly de-centralized. Health care and social care, including elderly care, are provided by different authorities on the regional and local level, respectively (see Additional file 1 for more detailed information about the study setting). The policy aimed to improve quality and coordination of care for the most ill elderly through supporting the development of local quality improvement capacity and strengthening the collaboration between health and social care. Key features of the policy were performance bonuses tied to the use of specific national quality registries (NQR), a new web portal providing real-time feedback of results, and funding for regional improvement coaches that were to support the implementation of NQRs and facilitate local quality improvement work. Halfway into the five-year implementation period, as a response to feedback on implementation progress, SALAR launched a program aiming to engage and support senior managers in regional implementation of the policy. SALAR coordinated the policy implementation on the national level and provided training and information. The policy also included requirements that the counties had to fulfill to be eligible for performance bonuses. The main policy components are shown in Table 1.

**Table 1:** The main components of the policy

Policy component	Content
Performance bonuses in five improvement areas	Improvement areas: 1. Preventive care (the Senior Alert Registry) 2. Palliative care (the Swedish Palliative Registry) 3. Dementia care (the Swedish Dementia Registry, SveDem; the BPSD Registry^a^) 4. Medical treatment 5. Coordinated care
Implementation support	• Real-time feedback of results • Regional improvement coaches • Senior management program (workshop series) • National coordination (SALAR)
Requirements for performance bonuses	• Collaborative management structures at the county level • Management system for systematic quality work

^a^BPSD – Behavioral and psychological symptoms in dementia

The policy agreement was renegotiated annually during the implementation period of 2010–2014, and the target levels for performance bonuses were successively increased. The policy has been described more thoroughly in previous publications [14, 16].

### Cases

The three counties were purposively selected to provide variation in geographical location, number of municipalities within each county and distribution of the population. County 1 was one of the largest regions in Sweden population-wise, including a metropolitan area. The county had one county council and 49 municipalities, which were divided into four administrative areas. County 2 was a middle-sized region with one county council and nine municipalities divided into three administrative areas, including both small cities and rural areas with close access to urban centers. County 3 was one of the largest regions in the country, considering the size of the territory, and 12 of the 15 municipalities were rural, sparsely populated areas. The county had one county council and was divided into three administrative areas: two areas, each encompassing a larger city, and a third area comprising sparsely populated municipalities.

### Data collection

Data used in this study was part of a larger project that covered the entire process of development and implementation of the policy between 2010 and 2014. Three types of data were used. Semi-structured telephone interviews with informants leading the implementation on county-level; i.e., improvement coaches and members of senior management teams (County 1, *n* = 6; County 2, *n* = 7; County 3, *n* = 5) were the main data source. In addition, county-level documents, such as actions plans, reports and descriptions of organizational structures (County 1, *n* = 13; County 2, *n* = 12; County 3, *n* = 14), national policy-related documents (*n* = 15), and observations of the national policy meetings and workshops (*n* = 8) were collected for the entire implementation period. The interviews were conducted in 2014. All of the informants were involved in the entire implementation process (between 2010 and 2014), and the interviews contained current and retrospective information about the past experiences. The interview guides were adapted to senior managers and improvement coaches respectively, while both versions covered the informants’ perceptions of the policy and implementation support, their roles, the organizational structures and communications and conditions and strategies for implementation. The interviews (30–60 min) were recorded and transcribed verbatim. The informants gave informed consent to participate before the interviews. In the project, a participatory research approach was used, and the researchers met regularly with participants to discuss preliminary results. The Regional Ethics Committee in Stockholm (ref no. 2011/5:11) judged that the study had no ethical aspects to be considered.

### Data analysis

The CFIR [8] guided the analysis of the interview data. A directed content analysis approach was used to cover the full variety in the data set [17]. Initially, the full range of domains and constructs was used during the coding process, but in the final analysis, four domains were found to contain the vast majority of the coded data (Table 2). The respondents did not touch upon all constructs; see Table 2 for operationalized descriptions of the constructs that were used in this study. The “Characteristics of the individuals” domain was not used, as the study focused on organizational level.

**Table 2:** Description of the CFIR domains and the operationalization of the constructs used in the study

Domains	Operationalization of the constructs and sub-constructs
*Intervention characteristics*	*Innovation Source*: Perception of key stakeholders about whether the innovation is externally or internally developed. *Evidence Strength and Quality*: Perceptions of the quality and validity of evidence supporting the belief that the innovation will have the desired outcomes. *Relative Advantage*: Perception of the advantages of implementing the policy versus alternative solutions, as well as advantages (or disadvantages) of the separate components. *Adaptability*: Perceived possibilities to adapt, tailor, refine or reinvent the policy to meet local needs. *Trialability*: The ability to test the policy content on a small scale in the organizations and to be able to “undo” implementation if warranted. *Complexity*: Perceived difficulty of the policy, reflected by duration, scope, radicalness and disruptiveness. *Design Quality and Packaging*: Perceived excellence in how the policy is bundled and presented; e.g., supporting materials and the overall composition of the policy.
*Outer setting*	*Needs and Resources of Those Served by the Organization*: The extent to which the needs of elderly with complex health needs are acknowledged by the organization. *Cosmopolitanism*: Communications and networks with actors and organizations external to the county and its health and social care organizations. *Peer Pressure*: Mimetic or competitive pressure to implement the policy in relation to county councils and municipalities in other counties. *External Policy and Incentives*: Perception of the policy as an external initiative and the performance bonuses as a policy instrument.
*Inner setting*	*Structural Characteristics*: The organizational structures for collaboration between actors in health and social care for elderly with complex health needs. *Networks and Communications*: The presence and quality of formal and informal communication and networks between health and social care for elderly with complex health needs. Implementation Climate *Tension for Change*: Perceived need for change regarding the policy goals. *Compatibility*: The fit between the policy and the current structures and workflows and the organization’s needs and values. *Relative Priority*: Shared perception of the importance of implementing the policy. *Organizational Incentives and Rewards*: Incentives tied to indicators within the policy improvement areas. *Goals and Feedback*: Monitoring policy goals and offering feedback to staff and managers. Readiness for Implementation *Leadership Engagement*: Commitment and involvement of leaders regarding the implementation of the policy. *Available Resources*: Resources available for local implementation. *Access to Knowledge and Information*: Ease of access to information, knowledge and support about how to implement the policy.
*Process*	*Planning*: The degree to which the policy goals are broken down and implementation activities are specified on local level. Engaging *Formally Appointed Internal Implementation Leaders*: Attracting and involving improvement coaches and members of senior management teams and their roles in the implementation.

First, text passages were deductively assigned to the relevant CFIR domains and constructs. Within each construct, meaning units were identified and given short descriptive codes. Second, codes were sorted into sub-categories. Two researchers were involved in the analysis. The first author conducted the main part of the analysis, but during each step, data samples from each county were coded in parallel by the first and second author and compared. Discrepancies were discussed, and the codes were revised based on consensus. Third, a memo was developed for each county, with summary statements and supporting quotes for each construct. The memos were used as a basis for describing and comparing the counties. Interview data was triangulated with information obtained from documents and observations.

## Results

### Characteristics of the intervention

Informants in all three counties had a clear picture of the policy as an *externally developed intervention*. Before the policy, the use of NQRs and local, data-driven systematic quality improvement in elderly care was limited in all three counties. The general view was that the NQRs were supported by sufficiently strong *evidence* and that there were definite *advantages* in using them. Also, access to real-time NQR results through the web portal, which was developed during the implementation, facilitated comparisons over time and between organizations and counties. The continuous feedback of results was seen as a central feature facilitating local quality improvement and a new possibility for senior managers and politicians to use up-to-date information as a basis for decisions. The improvement coaches were perceived as a key function needed to spur the development in all counties, both by adding competence about using the NQRs and data-driven quality improvement work, and as a link between health and social care.

The views differed regarding the senior management program. In County 1, it was described as an opportunity to develop collaboration. In County 2, a persisting view among the senior managers was that the existing collaborative structures were satisfactory and that there was little need to form new teams and participate in the senior management program. The improvement coaches, on the other hand, identified a need for greater management involvement in quality improvement. In County 3, the senior managers showed little interest in the program throughout the implementation. However, the policy still created external pressure, which was perceived as necessary to overcome inertia toward improving collaboration.

Often, when the issues are complex, it cannot be the individual special housing unit’s sole responsibility to try and solve the problem, but the county council has to do [its] part as well. At present, the policymakers are pushing the county council and municipalities to collaborate, and this is crucial … Had it not been for the policy, things would not have changed a bit. (County 3)

Some parts of the policy could be *adapted* to meet local needs, and this was appreciated by the counties, while other parts were perceived as less flexible. The improvement coaches’ support and the practical use of the NQRs were the components that could be adapted. In contrast, the requirements for participating in the senior management program were perceived as too detailed and difficult to meet, and the content of the program as too standardized. County 2, for example, found it hard to match the program’s requirements with its current inter-organizational collaborative teams.

I am sure that the forming of senior management teams has worked well in other counties, but it did not suit our methods. We already had our own established teams. [ … ] The policy’s demand for new teams has just made things more complicated for us. (County 2)

The policy was described as *complex*, as it comprised many components and involved substantial changes in work organization and management on several levels. As rapid, full-scale implementation of the NQRs was needed from the start to obtain performance bonuses, *trialability* (i.e. the ability to test the policy components on a small scale) was not perceived as relevant.

Informants in all three counties were positive about the *design and packaging* of the policy. The meetings, workshops, materials and tools were greatly appreciated and the project team at SALAR was described as credible and responsive. The storytelling approach that SALAR used in its communication about the policy was described as very helpful.

### Outer setting

Informants in the counties were well aware of *the need for improvement of the care for elderly* with complex health needs. As a coordinator of the policy implementation on national level, SALAR had a great influence on the counties’ *cosmopolitanism*—i.e., their networking with organizations on national level and in other counties. The informants were positive about SALAR’s efforts to provide new opportunities for learning and benchmarking regarding the policy issues.

This policy has really opened up the way for learning [from other organizations] across the country. The approach [that SALAR uses in communication about the policy] that there is neither time nor resources for reinventing the wheel makes everyone more prone to look at others’ results and methods and to draw on their experiences. (County 3)

However, these venues were also described as potential arenas for *peer pressure* mechanisms. Informants in all counties also mentioned self-initiated collaborations with neighboring county councils, municipalities and universities.

In general, the counties were positive about the concerted national efforts to implement the policy. However, a prevailing issue was the dilemma between the policy agreement as a steering instrument for the central government and the autonomy of the county councils and municipalities. Despite its soft law character, the policy was perceived as strong governance. A commonly held view was that though there were no formal sanctions for not implementing the policy, participation was not voluntary in practice.

What happens is that you tend to do what they [SALAR and the government] demand. [ … ] I mean, we ended up implementing Senior Alert even though it might not have been the best approach for us. However, we had to use it to get the performance bonuses. (County 3)

The informants described initial difficulties in coordinating a national policy with local plans and ongoing work, but at the same time, they acknowledged advantages of aligning local efforts with national initiatives.

It can be difficult to handle national initiatives that require coordination on a county level [ … ] It can lead to ambiguity if there are already ongoing projects on the local level that are not in line with the national initiative. Still, I think concerted efforts and national support are needed to make things happen. The policy creates pressure in a good way, like “Come on, let’s do this now and take advantage of the national support for working with these issues … ”. I also think it is important that everyone is doing it simultaneously. (County 1)

### Inner setting

The current *structures* for collaboration and the nature and quality of *networks and communications* between health and social care differed greatly between the counties. When the policy was launched, County 1 had well-functioning collaborative structures on the local level, but a less developed collaboration on the county level. County 2 had well-established, efficient collaborative structures on all levels. County 3 had collaborative structures on the county level, but informants reported that elderly care received relatively little focus in these forums compared to other issues. Moreover, collaboration on the local level was marked by a lack of agreement regarding responsibilities for patients with complex needs.

The financial *incentives*—i.e., performance bonuses—were based on indicators, which formed clear intermediate *goals* at an early stage in the implementation. Still, the *tension for change* varied between organizational levels and in relation to different policy components. The NQRs were not widely used before the policy but many care providers, particularly within municipal elderly care, warmly welcomed them. During the whole implementation period, the improvement coaches in all three counties regularly compiled and delivered *feedback*, mainly in the form of aggregated outcome data, to senior managers. At first, senior managers and administrative leaders did not express any explicit demands to use results from NQRs to monitor quality but gradually their interest grew, particularly in County 1 and 2. Over time, the increased awareness among managers and administrative leaders contributed to putting issues related to quality and coordination of care for the most ill elderly on the political agenda, though slightly less so in County 3. The perceived need to develop collaboration between health and social care also varied. County 1 needed to develop new collaborative structures on the regional level to be eligible for performance bonuses. In County 2 and 3, the tension for change regarding collaboration was weaker, but for different reasons. In County 2, the leadership perceived the current structures and processes to be satisfactory. In County 3, a long-standing tradition of poor collaboration and mistrust between the regional and local authorities inhibited the will to develop collaboration. Despite this, all three counties participated in the national implementation activities, including the senior management program.

The policy’s *compatibility* with current values and practices were similar in all three counties. The central values of the policy resonated with stakeholders on all levels within both health and social care from the start. However, early on it became clear some NQRs did not suit the ways of working for some care providers; e.g., hospitals in all three counties found one of the NQRs (Senior Alert) to be less compatible with acute care processes, as it focused on issues requiring long-term efforts, such as malnutrition and decubitus ulcers.

The performance bonuses, combined with the volume and intensity of SALAR’s efforts and the government’s active involvement in the implementation, eventually resulted in all three counties giving the policy high *relative priority*.

I think that the policy has brought attention to these issues for [local] politicians in a way that would not otherwise have occurred. When political agreements about these things are negotiated on a national level, it feels more natural for the local politicians to accept and engage in the decisions than if the propositions come from the local administrative officials. (County 2)

The *leadership engagement* varied between the counties. In County 1, upper and senior managers became actively involved in the development of the policy action plan. In County 2, the joint steering committee’s engagement was lower, as it prioritized other ongoing work addressing the policy areas. This persisting lack of endorsement of the policy made it more difficult for the improvement coaches to engage the care providers in using the NQRs. In County 3, the informants reported relatively weak engagement among politicians and upper and senior managers throughout the whole implementation period.

The policy required the county councils and the municipalities to allocate time and *resources* for the implementation; e.g., for staff to participate in training and for senior managers to participate in the workshops. Informants in County 3 reported that the rural municipalities found it difficult to meet these requirements due to the lack of manpower and the extremely small administrative organizations. In addition, the performance-based reimbursement system implied special challenges for several of the rural municipalities with strained budgets.

As a county council, we have more ‘muscles’ and more money. [ … ] We can spend the reimbursement in advance, so to speak. The rural municipalities, on the other hand [ … ] It is difficult for them. They do not have any extra resources to invest in advance … they simply cannot spend money they do not yet have and hope to get paid afterwards. (County 3)

All three counties reported good *access to policy-related information* training and tools, either online or via network meetings or through direct contacts with SALAR, during the whole implementation period.

### Process

All three counties developed action *plans* in line with the policy requirements, but the practical impact of these varied. In each county, improvement coaches and senior managers were *engaged* to implement the policy. Dedicated funding for improvement coaches was provided as part of the policy, and all three counties took the opportunity to hire coaches as internal implementation leaders. SALAR organized a national network to support and coordinate the coaches, which was active during the whole implementation period. The senior management teams were expected to champion the implementation of the policy in their regions. However, since no funding was offered for this, the counties’ approaches to the senior management program varied. County 1 acted in line with the policy’s intention and formed five senior management program teams, which were coordinated on the county level, and developed a policy action plan. In County 2, three senior management teams were formed when the program started, one for each administrative area, but due to upper management’s ambition to minimize the policy’s interference with current organizational processes, they had no practical function aside from representing the county at the program workshops. Instead, a small central administrative team was responsible for developing the county’s policy action plan and for monitoring results, mainly for the purpose of obtaining the performance bonuses. In County 3, only one single provisional senior management team was formed during the implementation period, due to the lack of interest among the senior managers. The members were mainly administrators without proper decision mandates—i.e., they were not senior managers—and the majority of the rural municipalities were not represented in the team at all. The team developed a policy action plan that had limited practical impact in the region.

In County 1, a regional coordinator was hired to support forms of collaboration between the improvement coaches and the senior management teams. In County 2 and 3 the connections between the improvement coaches and the senior management teams were weaker and less clear. As implementation progressed, however, new organizational solutions developed in County 2 which enabled the improvement coaches to facilitate the use of NQR data and quality improvement efforts on the senior management level.

We already have an established collaborative structure with work groups [ … ] but over time, the improvement coaches have come to play an important role in these groups by providing compiled information about results and analyses. Their roles and input have become well established in these groups. At the start, they were not included in these groups, but now they are key members. (County 2)

In County 3, the improvement coaches and the senior management team were somewhat out of sync with each other. The allocation of roles and functions between the coaches and the team remained more or less unclear during the entire implementation period.

I mean, first [the government] allocates funding to improvement coaches, who start to work on local level, building networks and developing plans for working toward the policy goals … And later, [it] adds requirements of a politically anchored policy action plan and makes a huge investment in senior management teams to lead the implementation of this plan. This caused some … maybe not conflict, but confusion, about the roles of the improvement coaches and the senior management team. (County 3)

## Discussion

In this study, we used the CFIR to investigate key actors’ perspectives on the implementation of a national health policy in three counties. In sum, they shared the view that the policy addressed important issues and all three counties participated in the implementation activities. Still, variation was found both within and between the counties regarding how the policy and the implementation were perceived and managed. Reasons for this variation were found in the interplay of factors related to the characteristics of the intervention, the inner and outer setting and the implementation process, which will be discussed below.

Improving health and social care for elderly with complex health problems can be described as a wicked issue. The multitude of actors in elderly care represents many different views on this issue and on possible solutions. The current national policy was a comprehensive and ambitious initiative. A main advantage was the flexible and innovative implementation strategies used to involve many actors and organizations on multiple levels, in both health and social care. To some extent, however, these strategies fell short in matching the complexity of the policy issue and the differences in the counties’ inner settings.

More specifically, some policy components, such as the improvement coaches and the NQRs, were readily accepted and implemented in the three counties. This successful implementation was facilitated by earmarked government funding and economic incentives for these parts of the policy, but also by a general shared understanding of the need and benefits of these components. Furthermore, these components were characterized by flexibility and adaptability. Flexibility and room for local adaptations are central elements in CFIR [8] and other models describing determinants for implementation e.g., [18–20].

Other components, such as the senior management program, evoked mixed responses. This policy component addressed truly complex issues, as it was launched to support the development of regional capacity to collaborate and to lead cross-organizational improvement initiatives. Despite this, the design of the senior management program lacked in flexibility and provided little support for local adaptations. SALAR urged the counties to form standardized cross-organizational teams that were expected to function in a similar manner in all counties. Thus, this particular policy component aimed to target a difficult issue in a complex system using rather generic solutions, which did not consider the unique circumstances of the counties. This resulted in only partial implementation of the component during the five-year implementation period. Despite variations in implementation success, the senior management program highlighted the gaps in cross-organizational collaboration in all three counties. This led to increased awareness among key actors in the counties, of specific problems that needed to be addressed. These results resonate with prior research suggesting that complex issues, such as developing organizational collaboration, can only be understood by trying to work out practical solutions [1, 21], and that collaborative strategies are well-suited to tackle wicked problems [22].

The senior management program mentioned above was also an example of how the current policy aimed to steer the local actions to a greater extent than most other previous (and contemporary) policies in the area. All three counties perceived the policy as more or less infringing on their autonomy and self-governance by prescribing specific forms for collaboration and demanding the use of specific national quality registries. Thus, from the county perspective, the policy challenged the statutory independence of the local authorities. Previous studies have highlighted similar problems associated with such non-coercive governance (soft laws) [23, 24]. Formally, such policy agreements imply that participation is voluntary, but in practice it is difficult for the local authorities to reject the policy, as it would involve potential loss of government funding. This type of policy also implies a shift in the policy actors’ roles, where the government is more operative than usual, and SALAR takes on a double role of being both client and contractor [23]. It has been argued that the use of such policy agreements represents a form of governance that supports a re-centralization of health care in Sweden [25].

One main component of the policy was the performance bonuses, i.e. funding obtained when certain goals were achieved. These bonuses were found to be a strong incentive for the counties. This is in line with previous research, suggesting that a policy “push” in terms of dedicated funding can increase the chances of successful implementation e.g., [20]. However, it is also important that goals for performance bonuses are realistically attainable [26]. We found that some organizations, particularly the rural municipalities in County 3, had difficulties allocating time and resources for managers to participate in development work and for training staff to use NQRs. This contributed to unwanted variation regarding the possibilities to reach the policy goals. Thus, there may be a risk that performance bonuses may preserve existing geographical inequalities in health and social care, or even create a downward spiral for less successful organizations due to lack of leverage [27, 28], which was contradictory to the policy goals.

Peer pressure mechanisms were also found to play an important role in the policy implementation. As mentioned earlier, SALAR is the joint interest organization of all county councils and municipalities in Sweden, and it has a strong position both among its members and as a national policy actor. SALAR’s expectations that all members would implement the policy, in combination with their role as a contracting partner in the policy agreement, created strong pressure on the counties. This is in line with research showing the importance of a mediating actor sharing the interests of both the government and the implementers on the regional and local levels, in bringing about compliance of soft laws [24]. Moreover, the establishment of networks (e.g., for improvement coaches) and regular network meetings were important parts of SALAR’s implementation strategies that enhanced contacts and fruitful exchange between organizations. However, these networks also formed arenas for benchmarking and competitive pressure in the implementation, as counties were expected to report implementation progress and share good examples with their peers [14, 16]. Continuous public reporting of results may also have added to the counties’ perceived pressure to implement the policy and to perform well [26].

The use of CFIR to investigate the implementation of this comprehensive, national policy was found to be beneficial for analyzing the rich data and for facilitating the comparisons across the counties. Previously, the CFIR was mainly used to study the implementation of fairly well-defined interventions in clinical settings [11]. A consequence of using the CFIR to study a county-level policy implementation was that the scope of the inner setting domain became broader, encompassing multiple organizational layers within several autonomous municipalities and a county council. Thus, as has been discussed previously, the CFIR could benefit from acknowledging the possibility of multiple organizational layers within the inner setting [12] when analyzing the implementation of more comprehensive interventions.

### Implications

The study has five main implications for formulating and implementing comprehensive policies to solve wicked problems. First, the need to form policy components that allow adaptations based on the local contextual factors is highlighted. The rather fixed format of the senior management program was more difficult to implement, as contrasted with the improvement coaches whose roles could be adapted. Second, the need for policies to balance between steering and self-governance in this type of decentralized health and social care system is emphasized. The senior management program was perceived as infringing the local authorities’ autonomy, but at the same time created external pressure to spur development. Third, there was variation within counties in how different types of actors interpreted the policy, which led to different prioritizations and complicated the implementation. This highlights the importance of using a collaborative approach to engage stakeholders with differing perspectives and tailoring policy communication to facilitate shared understanding and commitment. Fourth, counties’ inner settings may facilitate or hinder their opportunities to implement a policy. This illustrates the need for policymakers to carefully assess the target organizations’ implementation capacity and to be aware of the implicit quid pro quos involved in policy development. Finally, special attention is warranted regarding the use of financial incentives or other types of steering mechanisms in policies addressing wicked problems. The complexity of the issue makes it difficult to align incentives with the goals and to assess potential consequences.

### Methodological discussion

One of the strengths of this study was the use of an established meta-theoretical framework, the CFIR, for analyzing the data. One potential limitation of the study concerns the selection of the respondents and the issue of representativeness. The study focused on the implementation from the perspectives of key actors involved in the policy, and thus it made sense to interview those with long experience of the policy. However, at the end of the five-year policy implementation, due to staff turnover, there were rather few individuals who met the selection criteria for the study. It is possible that including key actors with less experience of the policy or informants not directly involved in managing the policy implementation (e.g., health and social care staff members or local politicians) would have generated another picture of the implementation in the three counties. However, they would not have been able to provide as comprehensive accounts of the implementation. The strength of the approach was that informants representing different perspectives were included. An additional strength was the possibility to triangulate the data across interviews, documents and observations. This was an attempt to balance the risk of individual informants having a vested interest in presenting overly positive perspectives of the local conditions and the implementation. Thus, the triangulation improved the internal validity of the study.

## Conclusions

The implementation of the policy components that addressed less wicked issues, and that could be adapted to local conditions, was perceived as feasible. In a similar manner, the component that targeted more complex problems and lacked in adaptability was perceived as more problematic. Performance bonuses and informal pressures exerted strong influence on the counties to implement the policy, despite poor fit between some of the policy components and the county contexts. From a county perspective, comprehensive health policies aiming to address wicked problems have better chances of succeeding if the implementation includes assessments of the target organizations’ implementation capacity as well as the implicit quid pro quos involved in policy development. Special attention is warranted regarding the use of financial incentives when dealing with wicked problems since the complexity makes it difficult to align incentives with the goals and to assess potential consequences. Other important aspects in the implementation of such policies are the use of collaborative approaches to engage stakeholders with differing perspectives, and the tailoring of policy communication to facilitate shared understanding and commitment. Finally, the CFIR was found to be a useful framework for investigating a comprehensive national policy and for comparing the three counties.

## Supplementary information

**Additional file 1.** Study setting. Information about the setting of the study.

## Data Availability

The datasets generated and analyzed during the current study are not publicly available due to the identifiable nature of our qualitative data but they are available from the corresponding author on reasonable request.
