# Supplementary material for: Implementation of a national policy for improving health and social care: a comparative case study using the Consolidated Framework for Implementation Research
Source: BMC Health Serv Res. 2019 Oct 22;19:730. doi: 10.1186/s12913-019-4591-2 (PMC6805604; doi:10.1186/s12913-019-4591-2)
Supplement: Supplementary file 1 — Additional file 1. Study setting. Information about the setting of the study. [file 12913_2019_4591_MOESM1_ESM.docx]

# Additional file 1

**Implementation of a national policy for improving health and social care: a comparative case study using the Consolidated Framework for Implementation Research**

Strehlenert, H., Hansson, J., Nyström, M. E. and Hasson, H.

## Study setting

The Swedish healthcare system is highly de-centralized. The responsibility is shared between the national government and self-governing regional and local authorities. In each of the 21 counties/regions a regional authority (county council) is responsible for financing and delivering both primary care and specialized health care. The county councils are subordinate to the national parliament, but they have a high degree of autonomy in organizing health care to meet the local needs (1). Also, within each county, several autonomous municipalities are responsible for financing and providing social care, including elderly care and home health care. Different laws regulate healthcare and social care respectively. Though the municipalities cover much smaller areas than the county councils, they are on equal footing in the legislation. Thus, collaboration between county councils and municipalities is necessary to provide coordinated, good quality care to elderly patients with complex needs (2).

The Swedish government rarely uses formal legislation to regulate matters concerning health care and social care; instead it typically relies on various forms of soft laws, such as national guidelines and policy agreements which can include financial incentives (3).

# References

1. Fredriksson M, Blomqvist P, Winblad U. Conflict and compliance in Swedish health care governance: Soft law in the “shadow of hierarchy.” Scan Polit Stud [Internet]. 2012 [cited 2015 Jan 14];35(1):48–70.

2. Anell A, Glenngård AH, Merkur S. Health Systems in Transition - Sweden Health system review. Health Syst Transit [Internet]. 2012;14(5):187.

3. Blomqvist P, editor. Vem styr vården? Organisation och politisk styrning inom svensk sjukvård. Stockholm: SNS förlag; 2007.
